# Lung ultrasound for fluid assessment in patients receiving dialysis—a systematic review

**DOI:** 10.1007/s40620-025-02435-x

**Published:** 2025-11-03

**Authors:** Nanna Aagaard Petersen, Anders Nikolai Ørsted Schultz, Casper Falster, Jan D. Kampmann

**Affiliations:** 1 Internal Medicine Research Unit, University Hospital of Southern Denmark, Aabenraa, Denmark; 2 Department of Respiratory Medicine, Odense University Hospital, Odense, Denmark; 3 Odense Respiratory Research Unit (ODIN), Department of Clinical Research, University of Southern Denmark, Odense, Denmark; 4 Department of Regional Health Research, University of Southern Denmark, Odense, Denmark

**Keywords:** Point of care ultrasound, Dialysis, Fluid assessment, Ultrafiltration

## Abstract

**Background:**

Accurate methods of assessing fluid status in patients with  kidney failure in need of dialysis are pivotal to preventing volume overload. Unidentified volume overload can lead to cardiac complications such as left ventricular hypertrophy, hypertension, and, ultimately, heart failure. Currently, there is no gold standard for determining the hydration status of this patient group. This systematic review aims to synthesise studies that have assessed the diagnostic accuracy of lung ultrasound in evaluating fluid status and the use of lung ultrasound to improve long-term endpoints in chronic dialysis patients.

**Methods:**

The protocol was registered in the PROSPERO registry (CRD 42022334147), and was conducted in accordance with PRISMA guidelines. The search was conducted in the PubMed, Embase, Medline and Web of Science databases to identify studies related to lung ultrasound and dialysis.

**Results:**

The search yielded 2543 studies, and after screening, five diagnostic accuracy studies and seven randomised controlled trials (RCTs) were included in the final analysis. The diagnostic accuracy studies reported sensitivity between 33 and 94.5% and specificity from 39 to 80%, highlighting considerable heterogeneity among the studies. The RCTs indicated that using ultrasound in volume management was not associated with improving long-term endpoints such as all-cause mortality and cardiovascular events. The quality assessment of the included studies showed an overall risk of bias in both types of studies (RCT and diagnostic accuracy studies).

**Conclusion:**

Lung ultrasound is an accurate, low-cost, and easily implementable method that could complement but not replace the standard of care. The available literature concerning the use of lung ultrasound in volume assessment of patients with kidney failure was characterised by heterogeneity and risk of bias. Future rigorous, high-quality research must involve randomised controlled trials to consolidate the efficiency and efficacy of lung ultrasound for kidney failure patients.

**Graphical abstract:**

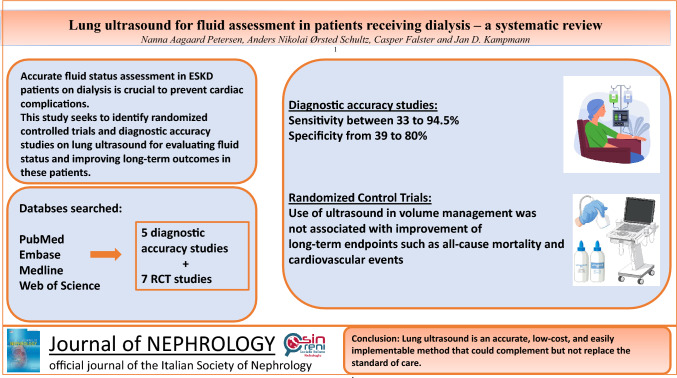

**Supplementary Information:**

The online version contains supplementary material available at 10.1007/s40620-025-02435-x.

## Introduction

Chronic kidney disease (CKD) affects 5–16% of the global population and is a leading cause of mortality worldwide [1, 2]. CKD is associated with cardiovascular disease (CVD), which may be aggravated as kidney function declines [3, 4]. As CKD progresses, patients with kidney failure may require kidney replacement therapy (KRT), the most common being hemodialysis (HD), followed by peritoneal dialysis (PD) [5] The global number of kidney failure patients is projected to reach 5,439,000 in 2030 [6]. In patients with CKD, especially those in need of KRT, volume overload is a common condition with a prevalence of up to 46% [7]. If not recognised promptly, volume overload can lead to cardiac complications such as left ventricular hypertrophy, hypertension, and, ultimately, heart failure [8]. Consequently, identifying accurate methods for assessing fluid status in dialysis patients is essential.

In the clinical setting, volume overload is often assessed through clinical examination, i.e. pedal oedema, lung crackles, blood pressure measurement and weight gain. However, these findings have limited diagnostic performance in relation to congestion [8, 9]. Although chest X-rays and bioimpedance analysis (BIA) techniques are widely used for predicting volume overload, these modalities are costly, time-consuming and lack bedside applicability [10]. Inferior vena cava diameter and collapsibility have been suggested as key parameters in echocardiographic fluid status assessment, indicating central venous pressure levels and potential renal congestion, but must be interpreted with consideration to factors like cardiac function, intrathoracic pressure, and intra-abdominal pressure [11].

Over the last decade, ultrasound has received increasing attention concerning its application for evaluating volume status in CKD patients. Ultrasound is non-invasive, radiation-free, allows point-of-care examination [12] and is characterised by low inter-operator variability [12]. Under certain conditions, its sensitivity and specificity exceed other diagnostic methods, such as cases of pleural effusion or inferior vena cava enlargement [13, 14]. Lung ultrasound has received specific scientific attention as an adjunct tool in the effort to achieve dry weight and improve clinical outcomes in patients with CKD [15].

A systematic review exploring the effect of lung ultrasound on fluid balance found that lung ultrasound reduced the cumulative fluid balance compared with conventional diagnostic modalities but did not consistently report other clinical outcomes, and only three of the twelve studies focused solely on nephrology patients [16]. Another systematic review explored lung ultrasound to evaluate the fluid status in HD patients and concluded that the level of B-lines was correlated with poor prognosis but did not report mortality [17]. Therefore, a synthesis of the literature concerning the diagnostic accuracy of lung ultrasound for PD patients and the use of lung ultrasound to improve other endpoints is highly warranted.

This systematic review aims to identify studies examining the clinical utility of lung ultrasound in evaluating fluid status or improving long-term endpoints for chronic dialysis patients.

## Methods

### Search strategy

The search strategy (Appendix 1) was created in collaboration with a research librarian. The protocol was registered with the PROSPERO registry (CRD 42022334147), and the study was conducted in accordance with Preferred Reporting Items for Systematic Reviews and Meta-analysis (PRISMA).

PubMed, Embase, Medline and Web of Science were searched for relevant literature between January 1 st, 2000 to November 29th, 2022.

### Study selection

Covidence [18] was used to remove duplicates and ensure double-blinded screening (NAP and JDK). If there were disagreements, a third reviewer (AS) was consulted to make a final decision. Eligibility criteria for lung ultrasound for fluid assessment in patients receiving HD- and/or PD are shown in Table 1. Studies were only included if they described an RCT or had a diagnostic accuracy design.

**Table 1:** Study characteristics

Author/Year/Country	Study design/Single-/multicenter	Outcomes	Comparison	Numbers included/Thereof Males/age	LUS score/machine/zones scanned	Main outcome and results
Alexiadris, G/2016/Greece	Observational study/Single center	Hydration status based on IVCD	BIA and Crit-line	53/34/63.4 ± 14.4 (SD)	none or mild < 14 BLS; moderate 14–30 BLS; severe > 30 BLS/General Electric Vivid 3 ultrasound unit/ 8 zones	**Predicting overhydration (by IVCDi)**: BLS: AUC = 0.81 (95% CI 0.74—0.87), Se 0.77 and Sp 0.74. BIA: AUC 0.71 (95%CI 0.63–0.78), Se 0.90, Sp 0.45; Crit-line: AUC 0.61 (95%CI 0.53–0.68), Se 0.82, Sp 0.39. **Predicting underhydration:** BLS: AUC 0.83 (95%CI 0.76–0.89), Se 0.78, Sp 0.73; BIA: AUC = 0.76 (95%CI 0.68–0.82), Se 0.71, Sp 0.80; Crit-line: AUC 0.53 (95%CI 0.44–0.61), Se 0.68, Sp 0.47
Allinovi, M/2022/Italy	Prospective observational/Single center	IDH (BP > = 20 mmHg and/or decrease in MAP > = 10 mmHg from pre-dialysis levels)	Physical examination, BVM, blood pressure, NT-proBNP and chest X-ray	107/35/69.1 (58.2–82.3 (median))	BLS ≤ 5 = euvolemia; BLS > 15 = hypervolemia/MyLab Class C-Esaote 6–18 MHz linear probe/28 zones	**Predication overhydration (by clinical evaluation and chest X-ray)**: Pre-dialysis BLS $\ge$ 15: BLS: Se 94.5%; Clinical evaluation alone: Se 72%; chest X-ray alone: Se 78%. ROC: AUC = 0.983 (95% CI 0.964–1.000) **Prediction the Risk of IDH:** ROC: AUC = 0.736 (95% CI 0.637–0.835)
Annamalai, I/2019/India	Cross sectional study/Single center	Hydration status based on HRCT	HRCT and IVCD	50/37/37.12 ± 10.8 (SD)	Mild 5–14 BLS; moderate 15–30 BLS; severe > 30 BLS. Clinically significant pulmonary congestion BLS ≥ 5/Aloka Prosound SSD-4000SV 7–12 MHz linear probe/28 zones	**Predicting overhydration (by HRCT)** BLS score of 7: Se 70% and Sp 80%. BLS of 12: of 92% for CT line score ≥ 20
Bobot, M/2020/France	Prospective observational/Single center	Dry weight reduction based on TTE	TTE and clinical score	31/22/63 [52–76] (quartile)	Mild 5–14 BLS; moderate 15–29 BLS; severe > 30 BLS/Philips1 CX50 POC, Amsterdam, Netherlands/28 zones	Diagnostic performance of lung ultrasound according to **TTE FO**: Se: 80% (95% CI: 38–96); Sp: 58% (95% CI: 39–75). Moderate to severe pulmonary overload (Echo comet-score > 15): Se: 80% (95% CI: 38–96), Sp: 62% (95% CI: 43–78). Severe pulmonary overload (ECS > 30): Se: 80% (95% CI: 38–96), Sp: 69% (95% CI: 50–84). Diagnostic performance of **clinical overload score** according to presence of pulmonary water on lung ultrasound: Se: 33% (95% CI: 15–58), Sp: 69% (95% CI: 44–86)
Loutradis, C/2021/Greece	Single-blinded randomized controlled trail/Multi center	Echocardiographic indexes of left ventricular (LV) mass functioning	Standard clinical treatment	Intervention group 35; control group 36/Intervention group 23; control group: 24/Intervention group 63.11 ± 13.52 (SD); control group 61.67 ± 13.67 (SD)	Dry-weight reduction: + history of CVD BLS ≥ 5; ÷ history CVD BLS ≥ 15/VScan device (GE, Horten, Norway)/28 zones	BLS: intervention group vs. control group (−4.34 ± 13.84 vs 4.94 ± 16.11; *P* = 0.004), paralleling dry-weight: intervention group vs. control group (−1.42 ± 2.47 vs 0.55 ± 2.33; *P* = 0.001). 12-month follow-up: LV end diastolic volume index: intervention group vs. control group (−0.94 ± 11.45 vs 6.58 ± 13.92 ml/m^2^; *P* = 0.015); LAVI: intervention group vs. control group (−3.22 ± 11.82 vs 4.76 ± 12.83 ml/m^2^; *P* = 0.009)
Loutradis, C/2019//Greece	Single-blind randomized clinical-trial/Multi center	Blood pressure monitoring and pulse wave velocity	Standard clinical treatment	Intervention group 35; control group 36/Intervention group 23; control group: 24/Intervention group 63.11 ± 13.52 (SD); control group 61.67 ± 13.67 (SD)	Dry-weight reduction: + history of CVD BLS ≥ 5; ÷ history CVD BLS ≥ 15/VScan device (GE, Horten, Norway)/28 zones	BLS: intervention group vs. control group (−2 [−7 to 0] vs 1 [0 to 3]; *p* < 0.001); dry-weight: intervention group vs. control group (−0.71 ± 1.39 vs + 0.51 ± 0.98 kg, *p* < 0.001). PWV: intervention group vs. control group (−0.25 ± 0.71 vs 0.20 ± 1.18 m/s p = 0.037); 48-h-central-pulse-pressure (cPP): intervention group vs. control group: (cPP 41.51 ± 9.63 vs 39.06 ± 9.61 mmHg; *p* = 0.004); 48-h-PWV: intervention group vs. control group (9.30 ± 2.00 vs 9.08 ± 2.04 m/s *p* = 0.032)
Loutradis, C/2018/Greece	Single-blind randomized clinical-trial/Multi center	48-houres blood pressure reduction	Standard clinical treatment	Intervention group 35; control group 36/Intervention group 23; control group: 24/Intervention group 63.11 ± 13.52 (SD); control group 61.67 ± 13.67 (SD)	Dry-weight reduction: + history of CVD BLS ≥ 5; ÷ history CVD BLS ≥ 15/VScan device (GE, Horten, Norway)/ 28 zones	BLS: intervention group vs. control group (–5.31 ± 12.53 vs. 2.17 ± 7.62, *P* < 0.001); dry weight: intervention group vs. control group (–0.71 ± 1.39 vs. 0.51 ± 0.98 kg, *P* < 0.001); 48-h SBP: intervention group vs. control group (–6.61 ± 9.57 vs. –0.67 ± 13.07, *P* = 0.033); DBP: intervention group vs. control group (−3.85 ± 6.34 vs. −0.55 ± 8.28, *P* = 0.031)
Loutradis, C/2019/Greece	Single-blind randomized clinical-trial/Multi center	Short-term BPV	Standard clinical treatment	Intervention group 35; control group 36/Intervention group 23; control group: 24/Intervention group 63.11 ± 13.52 (SD); control group 61.67 ± 13.67 (SD)	Dry-weight reduction: + history of CVD BLS ≥ 5; ÷ history CVD BLS ≥ 15/VScan device (GE, Horten, Norway)/ 28 zones	No significant changes in BPV during the 48-h period in both intervention group and control group (brachial SBP-ARV: 12.58 ± 3.37 vs. 11.91 ± 3.13, *P* = 0.117, brachial DBP-ARV: 9.14 ± 1.47 vs. 8.80 ± 1.96, *P* = 0.190)
Loutradis, C/2019/Greece	Single-blind randomized clinical-trial/Multi center	Echocardiographic indexes of left and right cardiac chamber size, as well as systolic and diastolic function	Standard clinical treatment	Intervention group 35; control group 36/Intervention group 23; control group: 24/Intervention group 63.11 ± 13.52 (SD); control group 61.67 ± 13.67 (SD)	Dry-weight reduction: + history of CVD BLS ≥ 5; ÷ history CVD BLS ≥ 15/VScan device (GE, Horten, Norway)/ 28 zones	BLS: intervention group vs. control group (−5.3 ± 12.5 vs + 2.2 ± 7.6; *P* < 0.001); dry weight: intervention group vs. control group (−0.71 ± 1.39 vs + 0.51 ± 0.98 kg; *P* < 0.001); IVC diameter: intervention group vs. control group (−0.43 ± 4.00 vs 0.71 ± 4.82 cm; *P* = 0.03); LV end-diastolic diameter: intervention group vs. control group (−4.97 ± 21.55 vs 1.59 ± 16.94 mm; *P* = 0.05): Left (LA) and right (RA) atrial dimensions: intervention group vs. control group (LA surface: −1.09 ± 4.61 vs 0.93 ± 3.06 cm^2^; *P* = 0.03; RA surface: −1.56 ± 6.17 vs 0.47 ± 2.31; *P* = 0.02)
Siriopol, D/2016/Romania	Randomized controlled trail/Multi center	All-cause mortality and first cardiovascular event (CVE)	Clinical evaluation of hydrations status	250/ 116/59.2 ± 14.1 (SD)	Moderate to severe ≥ 15 BLS pre-dialysis/n/a/n/a	Primary outcome: All-cause mortality and first CVE (HR 0 1.09, 95%CI 0.64–1.86, *p* = 0.75). Secondary outcome: all-cause mortality (HR = 1.02, 95% CI 0.53–1.96, *p* = 0.96) and CVE (HR = 0.89, 95% CI 0.49–1.59, *p* = 0.69)
Tan, G/2022/Singapore	Observational study/Single center	Performance of 8-point LUS using an HHUSD (handheld ultrasound device) (AI) in diagnosing fluid overload	BIA and clinical examination	76 (60 HD and 16 PD)/35/59 ± 11.0 (SD)	Normal 0–4 BLS; Lung congestion ≥ 5 BLS/Philips Lumify portable ultrasound unit equipped with a curved array transducer/8 zones	**Prediction of moderate to severe fluid overload** (by clinical examination): AUC 0.773 (95% CI 0.677–0.869, *p* < 0.0001). The optimal number of B lines > 4.5 (physician count) to predict overhydration Se 0.64, Sp 0.66
Zoccali, C/2021/Italy	Randomized controlled trail/Multi center	Heart failure, myocardial infarction and mortality	Standard clinical care	Intervention group 183; control group 180/intervention group 127; control group 128/intervention group 70 ± 10 (SD); control group 70 ± 11 (SD)	Mild < 15 BLS; severe > 15 BLS/Handheld US machine (VS scan, General Electric)/28 zones	Treatment target (< 15 US-B lines): intervention group arm vs. control group (78% vs. 56%, *P* < 0.001). Primary outcome: composite of all-cause death, non-fatal myocardial infarction, decompensated heart failure: HR: 0.88, 95% CI: 0.63–1.24, *P* = 0.47. Secondary outcome (echocardiographic parameters, risk for all- cause and cardiovascular hospitalizations): all-cause hospitalizations: HR: 1.03; 95% CI: 0.77–1.36, *P* = 0.86; cardiovascular hospitalizations: HR: 1.02, 95% CI: 0.71–1.46, *P* = 0.92. Post hoc analysis: repeated episodes of decompensated HF (IRR = 0.37, 95%CI 0.15–0.93, *p* = 0.035) and repeated cardiovascular events (IRR = 0.63, 95% CI 0.41–0.97, *p* = 0.038)

*IVCD* inferior vena cava diameter, *BIA* bioelectrical impedance analysis, *BLS* B-line score, *AUC* area under the curve, *Se* sensitivity, *Sp* specificity, *ROC* receiver operating characteristic curve, *BP* blood pressure, *SBP* systolic blood pressure, *DBP* diastolic blood pressure, *MAP* Mean Arterial Pressure, *BVM* blood volume management, *IDH* intradialytic hypertension, *TTE* Transthoracic echocardiography, *HRCT* high resolution CT, *LAVI* left atrial volume, *PWV* Office- pulse-wave-velocity, *BPV* blood pressure variability, *ARV* Abbreviations, *CVE* cardiovascular events, *HR* Hazard ratio, *IRR* incidence rate ratio

Scription in bold: outcome measures in diagnostic accuracy studies

Exclusion criteria were studies published in languages other than English, Danish, Swedish or Norwegian. Published commentaries, editorials, case reports and reviews were also excluded.

### Data collection, bias and quality assessment

Two authors (NAP and JDK) extracted data independently using a data extraction form first piloted with five studies. The data extracted for each study included study design, single- or multicentre study, outcome measures, comparison with the gold standard, dialysis modality (HD and/or PD), number of participants, gender, age and comorbidities, number of ultrasound scans, ultrasound machine used, lung zones, B-line scores, ultrasound operator and level of expertise including inter-observer reliability.

The Quality Assessment of Diagnostic Accuracy Studies-2 (QUADAS-2) score [19, 20] and revised Cochrane risk-of-bias tool (RoB 2) were used independently by two investigators (NAP and JDK) to assess diagnostic accuracy and RCT studies, respectively. The heterogeneity of study populations and designs resulted in a primarily narrative synthesis.

## Results

**Fig. 1:**
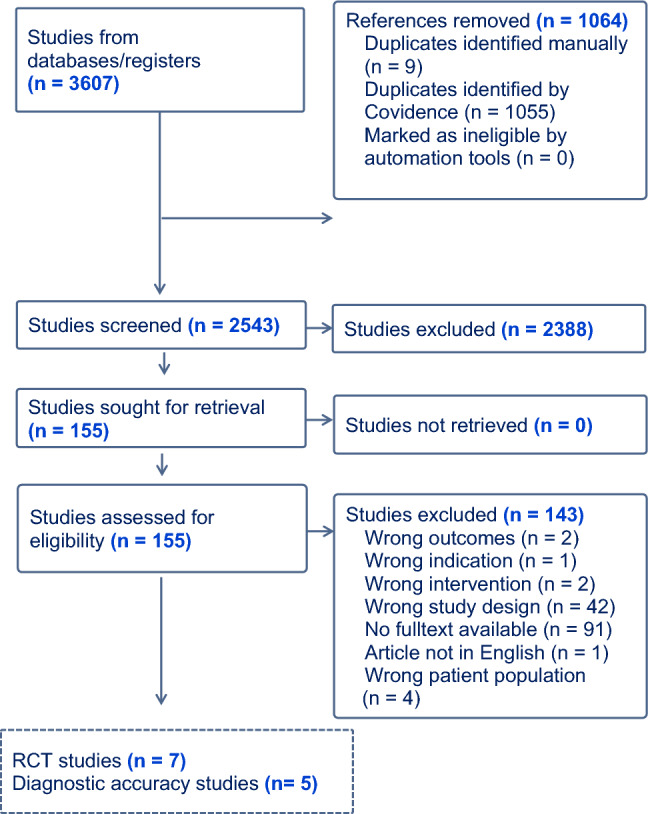
Study flow

The search yielded 2543 studies, 155 studies were eligible for full-text assessment, with 12 studies included in the final synthesis (5 diagnostic accuracy studies and 7 RCTs) (see Fig. 1).

### Diagnostic accuracy studies

Five studies reported the diagnostic accuracy of ultrasound-guided assessment of pulmonary congestion [21–25]. Findings for each study are reported narratively below (Table 1).

Alexiadis et al. [24] investigated the discriminative power of B-line scores calculated by lung ultrasound in eight zones compared to bioimpedance analysis and Crit-line (hematocrit and oxygen saturation) for predicting over- and underhydration, determined by Indexed inferior Vena Cava Diameter at inspiration (IVCDi). Lung ultrasound was performed with a General Electric Vivid 3 ultrasound unit, and lung congestion was defined as none or mild if B-line score < 14, moderate if the B-line score was 14–30 and severe if B-line score > 30. B-line score was reported as a better predictor of overhydration with a sensitivity and specificity of 77% and 74%, respectively, with an optimal cut-off when B-line score $\ge$ 11, compared to bioimpedance analysis (sensitivity of 90% and specificity of 45%) and Crit-line (sensitivity 82% and specificity 39%). B-line score was a better predictor for diagnosing underhydration, with a sensitivity of 78% and specificity of 73%, compared to bioimpedance analysis (71% and 80%) or Crit-line (68% and 47%).

Annamalai et al. [22] investigated the discriminative power of lung ultrasound using an Aloka Prosound 7–12 MHz linear probe in 28 zones to detect pulmonary congestion with high-resolution computed tomography (CT) scans. Lung congestion was defined as mild if the B-line score was 5–14, moderate if it was 15–30, and severe if > 30. Clinically significant pulmonary congestion was assumed if the B-line score ≥ 5. Annamalai et al. (2022) reported a sensitivity of 70% and specificity of 80% for a B-line score of 7 in detecting CT-lines > 20. B-line scores > 12 had a higher specificity (92%) for CT lines $\ge$ 20.

Bobot et al. [25] investigated the diagnostic performance of lung ultrasound using a clinical score for fluid overload and transthoracic echocardiography as the gold standard. Lung ultrasound was performed with a Philips1 CX50 POC in 28 zones with lung congestion defined by a B-line score 5–14 as mild, 15–29 as moderate and $\ge 30$ as severe. For detecting mild to severe pulmonary congestion, lung ultrasound (B-line score 5 to > 30) had a sensitivity of 80% and a specificity of 58%. The specificity increased to 62% when only moderate to severe overload (B-line score 15 to > 30) was examined, and to 69% in severe overload alone (B-line score > 30), compared with the clinical overload score (sensitivity 100% and specificity 77%).

Allinovi et al. [23] investigated the diagnostic accuracy of lung ultrasound in detecting and predicting episodes of intradialytic hypotension. Lung ultrasound was performed using a MyLab Class C-Esaote 6–18 MHz linear probe in 28 zones with a B-line score ≤ 5 indicating euvolemia and a B-line score > 15 reflecting hypervolemia. Lung ultrasound was reported to be a better predictor of fluid overload (defined through clinical examination and chest X-ray) with a sensitivity of 94.5% compared to clinical evaluation and chest X-ray alone (72% and 78%, respectively). In predicting episodes of intradialytic hypotension, an increase in one B-line resulted in lower odds (odds ratio [OR] 0.946 95% confidence interval [CI] 0.913–0.979, *p* = 0.003) of experiencing an episode of intradialytic hypotension. In patients who experienced intradialytic hypotension, B-line scores post dialysis were low (OR 0.895, 95% CI 0.7830–0.9684) with an optimal threshold of five B-line scores when patients had experienced intradialytic hypotension.

Tan et al. [21] investigated the diagnostic accuracy of lung ultrasound performed in eight zones using a Philips Lumify portable ultrasound unit equipped with a curved array transducer compared to clinical examination and bioimpedance analysis. This was the only study that included HD (60 patients) and PD patients (16). Lung congestion was defined as a B-line score ≥ 5, whereas a B-line score 0–4 was defined as normal. They found the optimal number of B-lines to be 4.5 in predicting fluid overload with BIA as a reference test, with a sensitivity and specificity of 74% and 76%, respectively.

### Randomised control trials

Seven RCTs were included in the synthesis. Notably, five of these studies reported on the same study population but described different endpoints [26–30].

The LUST trial [31] was a multicentre trial involving 18 renal units, with 367 enrolled patients on HD (183 in the intervention group, 180 in the control group) with a high-risk cardiovascular profile (history of myocardial infarction or heart failure (HF)). Patients were randomised to standard care or lung ultrasound-guided treatment. Lung ultrasound was performed before and after an HD session. In the intervention group, the number of patients who achieved the treatment target of a B-line score < 15 was higher (78%) compared to the control group (56%) (*p* < 0.001). Primary outcome (composite of all-cause death, non-fatal myocardial infarction, decompensated heart failure) did not differ between the intervention and control groups (*p* = 0.47). Secondary endpoints, including echocardiographic parameters, risk of all- cause hospitalisations (*p* = 0.86), and cardiovascular hospitalisations (*p* = 0.92) were similar in the two groups. However, there was a significant reduction between recurrent episodes of decompensated HF (*p* = 0.035) and cardiovascular events (*p* = 0.038) in the intervention group.

Loutradis et al. enrolled 71 HD patients with hypertension who were clinically euvolemic. Of these, 28 patients (39%) were also included in the LUST trial (Zoccali et al. 2021). Patients were randomised for dry weight reduction guided by pre-HD lung ultrasound and standard care treatment. Results showed a significant (*p* < 0.0001) intensification in ultrafiltration in the intervention group (54%) compared to the control group (14%). Considering echocardiographic parameters, inferior vena cava diameter at inspiration (*p* = 0.03) and left arterial surface (*p* = 0.03) decreased in the intervention group compared with the control group [28]. At the 12-month follow-up, the left ventricular end-diastolic volume (*p* = 0.015), left atrial surface (*p* = 0.006), and volume index (*p* = 0.009) decreased in the intervention group compared to the control group [30]. Secondary results showed a significant reduction in 48-h blood pressure (*p* = 0.033), B-line score (*p* = 0.017), dry weight (*p* = 0.005) [27], and arterial stiffness (*p* = 0.037) (expressed as pulse-wave velocity) in the intervention group compared to the control group [26]. However, diastolic function increased in the control group (*p* = 0.003) [29].

Siriopol et al. [32] investigated 250 patients on HD with low cardiovascular risk, randomised to dry weight adjustments by clinical criteria alone or lung ultrasound combined with BIA. The primary outcome was a composite of all-cause mortality and first cardiovascular event. The study indicated no significant difference between the active and control group (*p* = 0.75). Secondary outcomes of all-cause mortality and first cardiovascular event showed no significant difference (*p* = 0.96 and *p* = 0.69, respectively). Furthermore, the rates of intradialytic hypotension, all-cause hospital admissions, and vascular access thrombosis were not significantly different between the two groups.

### Quality assessment

Quality assessment in the five diagnostic accuracy studies showed that four out of five studies had a risk of bias in at least one domain (see Supplementary 1). Overall, quality assessment showed unclear judgement regarding the index test (60% of the studies) and patient selection (60% of the studies) (Table 2). According to risk of bias in the reference standard, one study (20%) had a high risk of bias, whereas the remaining studies indicated a low risk (80%) of bias. Concerns regarding the application of the overall judgement showed low levels of concern.

**Table 2:**
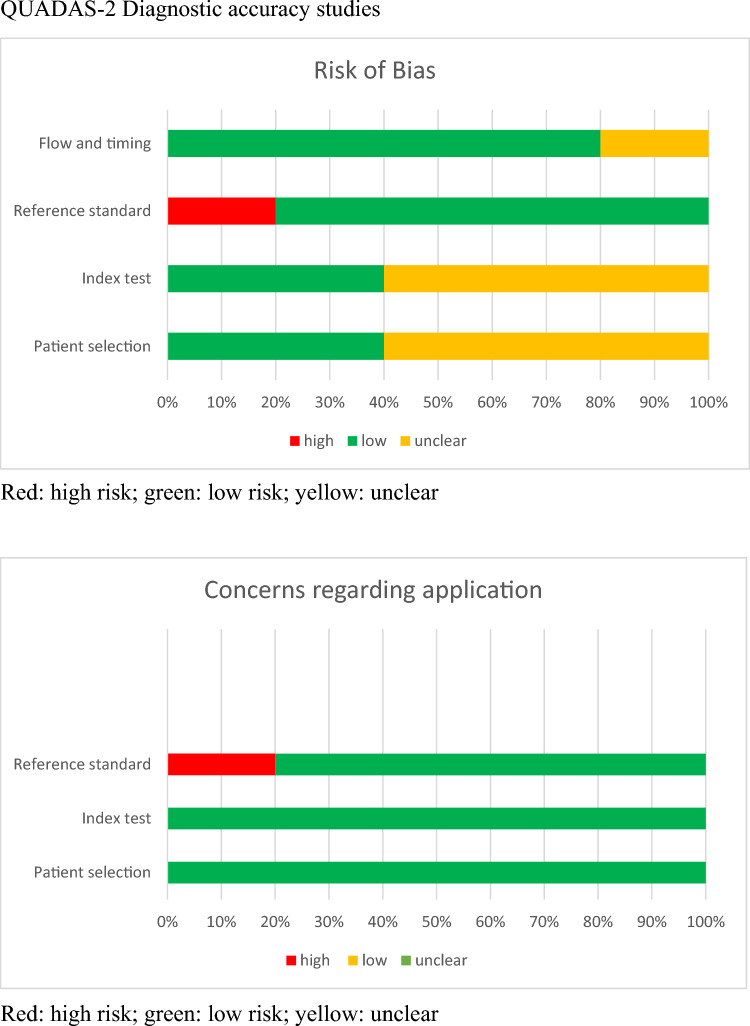
QUADAS-2 diagnostic accuracy studies

Quality assessment of the seven RCTs showed similar results except for the risk of bias arising from the randomisation process, which raised some concerns (see Supplement 1). An important source of potential bias was the lack of blinding to treatment assignment, which increased the conclusion to “some concerns” in the overall risk of bias of all included RCT studies despite different attempts to minimise this concern.

## Discussion

This systematic review identified five diagnostic accuracy studies and seven RCTs reporting on the clinical utility of lung ultrasound in volume management of patients with kidney failure.

Most excluded studies focused exclusively on children or did not use ultrasound as the examination method. A lack of blinding and small patient samples for the RCTs limited study quality. Five studies investigated the diagnostic accuracy of lung ultrasound for volume assessment in patients undergoing HD [21–25] or PD [21]. As there is only one study with few patients focused on PD, we still lack sufficient knowledge about PD patients and lung ultrasound.

Lung ultrasound was found to have reasonably good sensitivity and specificity in detecting overhydration defined by transthoracic echocardiography, inferior vena cava diameter at inspiration, as well as high-resolution CT. It provided better accuracy than bioelectrical impedance analysis, clinical examination, and X-ray. Naturally, as there is no generally accepted gold standard regarding overhydration, the diverse reference standards introduce bias. This point is exemplified in the literature describing diagnostic accuracy studies of inferior vena cava diameter at inspiration. One systematic review [33] concluded that inferior vena cava diameter at inspiration was unreliable for fluid prediction in populations containing healthy and critically ill patients. Studies have also shown a lack of sensitivity and specificity in diagnosing clinical examinations of over- and underhydration in healthy individuals [34]. Despite attempts to define a gold standard, no current methods have proven adequate.

Three studies [22, 23, 25] used an ultrasound regimen of 28 zones, whereas two studies [21, 24] used an 8-zone protocol. Torino et al. [35] included 303 HD patients, comparing 8 zones vs 28 zones, the 8-site score was in good agreement with a diagnosis of mild, moderate, and severe lung congestion and is a faster approach in everyday clinical practice (1.3 min vs 3.0 min) compared to the standard 28-site scan [35]. Although Annamalai et al. [22] and Bobot et al. [25] performed 28-zone lung ultrasound using the same lung ultrasound score, we opted not to perform a meta-analysis due to the bias assessed in the Bobot et al. reference standard [25, 36].

Regarding patient selection, there was a diversity in exclusion criteria, with Annamalai et al. [22] excluding patients with New York Heart Association (NYHA) Class III and IV and Alexiadris et al. [24] excluding patients with NYHA Class IV due to the impact of advanced heart failure on the number of B-lines. Despite this risk, approximately 74% of patients with CKD have heart failure, with increased mortality in this population [37]. Lung ultrasound is a reliable tool in blood pressure regulation through dry weight reduction [23, 27] and a prognostic factor for cardiovascular events and mortality [38, 39], so patients with a higher NYHA score may benefit from a more accurate dry weight reduction. Interestingly, excluding these patients may result in data from a patient group that is less prone to confounding; however, including them could provide valuable prognostic information on a particularly vulnerable patient group.

Only two studies [23, 25] reported that physicians performing the lung ultrasound examination were blinded to the results of the reference standard. This is an important bias as the interpretation of ultrasound findings may be influenced by the physician having any preconceived knowledge. There was only one study [21] that evaluated the interrater reliability of the ultrasound investigators. Tan et al. [21] reported a moderate inter-rater reliability with a Cohen’s kappa of 0.503 (*p* < 0.001), which is in concordance with Liang et al. [40], with reports of an inter-observer variability of 0.725 (*p* = 0.05).

With regard to the diagnostic accuracy studies, many studies applied different index and reference tests, making a comparison unfeasible, and there was a lack of blinding in the interpretation of the reference standard. However, applicability was low due to the subjectivity of the interpretation of the ultrasound analysis, as shown by Anderson et al. [33]. Indeed, the reported sensitivity spanned from 33 to 94.5% and specificity from 39 to 80%, leaving the question of whether lung ultrasound should be a standard tool for predicting fluid status in HD patients unanswered. Seven studies compared lung ultrasound with standard care in patients undergoing HD. Notably, five studies reported on the same study population but with different endpoints [26–30]. Considering hard endpoints, there was no difference between the two groups in all-cause mortality or cardiovascular events regardless of high [31] or low cardiovascular risk [32]. However, improvements in echocardiography parameters were seen in patients when ultrasound was used to determine ultrafiltration [28].

In an ad hoc analysis, Loutradis et al. reported a reduction in blood pressure, an improvement of diastolic functioning according to echocardiography [28], and an overall intensification of ultrafiltration in patients with lung ultrasound management. These authors proposed the lung ultrasound-guided strategy as an aid for blood pressure control, better dry-weight achievement, and reduction in adverse cardiac changes in this population [26].

When interpreting the results of RCTs, it should be considered that Zoccali [31] and Siriopol [32] did not reach their pre-specified sample sizes, resulting in a lack of power and an increased risk of type 1 and 2 errors. However, Loutradis et al. [26] included 71 patients, providing sufficient power to detect differences of 7 mmHg between groups in the 48-h systolic blood pressure change.

Limitations in interpreting the RCTs include a lack of homogeneity and a high risk of bias. Moreover, the studies were small, and five [26–30] included 39% of the same patient population as Zoccali et al. [31]. Further, most of the studies (60%) reported ultrafiltration as the outcome measurement. However, ultrafiltration does not provide the same accuracy in controlling fluid status in PD patients, resulting in transferability problems between the different dialysis modalities. This problem is also highlighted in a recent review by Kharat et al. [41]].

In conclusion, lung ultrasound is a precise, cost-effective, and easily implementable technique that can complement, but not replace, standard care. It is important to highlight that further studies are needed to establish a gold standard for volume assessment in patients with CKD. Some research indicates that lung ultrasound could be beneficial in this context, particularly regarding efficiency; however, there is no evidence linking it to all-cause mortality or cardiovascular events. More rigorous, high-quality research, including randomised controlled trials, are necessary to confirm lung ultrasound efficiency and efficacy. Current data are limited by their descriptive nature, and the analysed samples often consist of small patient groups with varying outcomes and biases.

## Supplementary Information

Below is the link to the electronic supplementary material.

Supplementary file1 (DOCX 17 KB)

Supplementary file2 (XLSX 15 KB)

## Data Availability

Date available on request.
