# Supplementary material for: Lung ultrasound for fluid assessment in patients receiving dialysis—a systematic review
Source: J Nephrol. 2025 Nov 3;38(9):2557–70. doi: 10.1007/s40620-025-02435-x (PMC12712114; doi:10.1007/s40620-025-02435-x)
Supplement: Supplementary file 1 — Supplementary file1 (DOCX 17 KB) [file 40620_2025_2435_MOESM1_ESM.docx]

**Appendix 1**

Search strategy

Ovid MEDLINE(R) ALL <1946 to May 20, 2024>

1                           Ultrasonography/                      203858

2                           echogram*.ti,ab,kf.                  803

3                           echograph*.ti,ab,kf.                 10498

4                           echoscop*.ti,ab,kf.                   135

5                           echosound*.ti,ab,kf.                133

6                           sonogram*.ti,ab,kf.                  3929

7                           sonograph*.ti,ab,kf.                 61816

8                           echotomograph*.ti,ab,kf.      764

9                           ultraso*.ti,ab,kf.                        484622

10                        1 or 2 or 3 or 4 or 5 or 6 or 7 or 8 or 9             584724

11                        Lung/                 257945

12                        Pleura/              10620

13                        Thorax/             23760

14                        lung*.ti,ab,kf. 851067

15                        pleur*.ti,ab,kf.                            95581

16                        thora*.ti,ab,kf.                           259016

17                        chest*.ti,ab,kf.                            224621

18                        pulm*.ti,ab,kf.                            668460

19                        pneu*.ti,ab,kf. 355147

20                        11 or 12 or 13 or 14 or 15 or 16 or 17 or 18 or 19                   1947452

21                        Renal Dialysis/ 104478

22                        Hemodiafiltration/                    2670

23                        Continuous Renal Replacement Therapy/     925

24                        Hemodialysis, Home/               2204

25                        Peritoneal Dialysis/                    20366

26                        Dialysis/            12822

27                        Renal insufficiency, Chronic/ 39025

28                        (Hemodia* or haemodia*).ti,ab,kf.                 95532

29                        Renal replacement therap*.ti,ab,kf.               18611

30                        dialys*.ti,ab,kf.                           136069

31                        Renal insufficienc*.ti,ab,kf.   25715

32                        CVVHDF.ti,ab,kf.                        399

33                        (ESRF or ESKF or ESRD or ESKD).ti,ab,kf.        24377

34                        (CKF or CKD or CRF or CRD).ti,ab,kf.                69546

35                        (CAPD or CCPD or APD).ti,ab,kf.                        13211

36                        21 or 22 or 23 or 24 or 25 or 26 or 27 or 28 or 29 or 30 or 31 or 32 or 33 or 34 or 35        324847

37                        10 and 20 and 36                        722

38                        limit 37 to yr="2000 -Current"                           660

Embase Classic+Embase <1947 to 2024 May 20>

1                           echography/   405263

2                           echogram*.ti,ab,kf.                  1204

3                           echograph*.ti,ab,kf.                 14390

4                           echoscop*.ti,ab,kf.                   196

5                           echosound*.ti,ab,kf.                136

6                           sonogram*.ti,ab,kf.                  5461

7                           sonograph.ti,ab,kf.                    146

8                           echotomograph*.ti,ab,kf.      982

9                           ultraso*.ti,ab,kf.                        728304

10                        1 or 2 or 3 or 4 or 5 or 6 or 7 or 8 or 9             917671

11                        lung/                  272309

12                        pleura/              16381

13                        thorax/              95883

14                        lung*.ti,ab,kf. 1287311

15                        pleur*.ti,ab,kf.                            151778

16                        thora*.ti,ab,kf.                           400819

17                        chest*.ti,ab,kf.                            388752

18                        pulm*.ti,ab,kf.                            1011479

19                        pneu*.ti,ab,kf. 527201

20                        11 or 12 or 13 or 14 or 15 or 16 or 17 or 18 or 19                   2866796

21                        exp hemodialysis/                     150236

22                        hemofiltration/                           8007

23                        Continuous renal replacement therapy/       9500

24                        Home dialysis/                             3826

25                        Peritoneal dialysis/                    44088

26                        Dialysis/            72172

27                        End stage renal disease/         57090

28                        (hemodia* or haemodia*).ti,ab,kf.                  148868

29                        renal replacement therap*.ti,ab,kf.                33338

30                        dialys*.ti,ab,kf.                           212866

31                        Renal insufficienc*.ti,ab,kf.   40736

32                        CVVHDF.ti,ab,kf.                        936

33                        (ESRF or ESKF or ESRD or ESKD).ti,ab,kf.        45699

34                        (CKF or CKD or CRF or CRD).ti,ab,kf.                121759

35                        (CAPD or CCPD or APD).ti,ab,kf.                        18657

36                        21 or 22 or 23 or 24 or 25 or 26 or 27 or 28 or 29 or 30 or 31 or 32 or 33 or 34 or 35        529412

37                        10 and 20 and 36                        2579
